# Mapping of quantitative trait loci associated with resistance to net form net blotch (*Pyrenophora teres* f. *teres)* in a doubled haploid Norwegian barley population

**DOI:** 10.1371/journal.pone.0175773

**Published:** 2017-04-27

**Authors:** Ronja Wonneberger, Andrea Ficke, Morten Lillemo

**Affiliations:** 1 Department of Plant Sciences, Norwegian University of Life Sciences, Ås, Norway; 2 Division for Biotechnology and Plant Health, Norwegian Institute of Bioeconomy Research, Ås, Norway; CIRAD, FRANCE

## Abstract

Barley net blotch caused by the necrotrophic fungus *Pyrenophora teres* is a major barley disease in Norway. It can cause grain shriveling and yield losses, and resistance in currently grown cultivars is insufficient. In this study, a set of 589 polymorphic SNP markers was used to map resistance loci in a population of 109 doubled haploid lines from a cross between the closely related Norwegian cultivars Arve (moderately susceptible) and Lavrans (moderately resistant). Resistance to three net form net blotch (*P*. *teres* f. *teres*) single spore isolates was evaluated at the seedling stage in the greenhouse and at the adult plant stage under field conditions during three years. Days to heading and plant height were scored to assess their influence on disease severity. At the seedling stage, three to four quantitative trait loci (QTL) associated with resistance were found per isolate used. A major, putatively novel QTL was identified on chromosome 5H, accounting for 23–48% of the genetic variation. Additional QTL explaining between 12 and 16.5% were found on chromosomes 4H, 5H, 6H and 7H, with the one on 6H being race-specific. The major QTL on 5H was also found in adult plants under field conditions in three years (explaining up to 55%) and the 7H QTL was found in field trials in one year. Additional adult plant resistance QTL on 3H, 6H and 7H were significant in single years. The resistance on chromosomes 3H, 5H, 6H and 7H originates from the more resistant parent Lavrans, while the resistance on 4H is conferred by Arve. The genetic markers associated with the QTL found in this study will benefit marker-assisted selection for resistance against net blotch.

## Introduction

The necrotrophic fungus *Pyrenophora teres* Drechsler (anamorph *Drechslera teres* (Sacc.) Shoemaker) is the causal agent of net blotch (NB), a foliar disease on barley, which occurs predominantly in cool and humid barley growing regions around the world [1]. Yield losses up to 44% have been reported under conducive conditions [2]. There are two forms of the pathogen, *P*. *teres* f. *teres and P*. *teres* f. *maculata*, which cause net form net blotch (NFNB) and spot form net blotch (SFNB), respectively. The stubble-born disease has been on the rise globally with the increased usage of reduced tillage practices in recent years [3]. In Norway, NB is an important barley disease with varying severity in different years and regions [4], but yield losses due to *P*. *teres* are not well-documented and reliable data is lacking. Both forms of the pathogen are found in Norway but it is not known which one is the dominant form.

Controlling the disease by the use of resistant varieties is desirable, but at present resistance of Norwegian cultivars is insufficient. All currently grown Norwegian cultivars are susceptible, moderately susceptible or moderately resistant to NB. Under these circumstances, crop rotation, tilling and pesticide treatment are the only effective means to control the disease. Resistance breeding is a more sustainable measure to reduce yield losses due to NB and even small increments in resistance will complement and enhance the effects of other control measures. More in-depth knowledge of this host-pathogen interaction will be of great benefit for resistance breeding.

Resistance to NB is usually governed by multiple genes, and several different resistance mechanisms are present in the pathosystem. Resistance can be dominant [5–7], recessive [8, 9] or incompletely dominant [10], and both major genes and minor effect quantitative trait loci (QTL) can be involved (reviewed in [11]). QTL harboring resistance to NB have been found on all chromosomes [11]. Since resistance to NFNB and SFNB is inherited independently [11, 12] and the two forms are genetically distinct, it is important to investigate both diseases separately. As is the case for many diseases, resistance to NB depends on the developmental stage of the plant. Some resistance QTL are only found in seedlings or adult plants, while others are reported to be associated with resistance at all stages [12–14]. Resistance under field conditions is often more complex than in seedlings tested under greenhouse conditions [15], in addition to being dependent on environmental conditions during the growth season and inoculum concentration [16]. A promising approach to breeding for long-lasting polygenic resistance is thus to pyramid different genes effective in seedlings and adult plants and against a wide range of isolates of both forms of the pathogen.

To our knowledge, this is the first QTL mapping study of resistance against NB in Norwegian cultivars. A biparental mapping population of 109 doubled-haploid lines segregating for NB resistance was created from a cross of the moderately susceptible cultivar ‘Arve’ and the moderately resistant cultivar ‘Lavrans’. Arve and Lavrans were widely grown during the 1990s and 2000s and are parents to some of the cultivars grown currently in Norway. Arve was previously characterized as highly susceptible to net blotch whereas Lavrans possessed moderate resistance [17](M. Lillemo, pers. comm.). Even though the susceptibility of both cultivars has changed since their release, Lavrans has always been consistently more resistant than Arve, which indicates that resistance in Lavrans is likely race non-specific. The population was tested for adult plant resistance under field conditions in inoculated and mist-irrigated hillplots over three years and for seedling resistance under greenhouse conditions. The objectives of this study were (1) to identify and map QTL associated with resistance to NB in Norwegian barley cultivars, (2) to test whether these QTL are stable throughout different environments, years and developmental stages and (3) to assess whether resistance screenings at the seedling stage can be used to predict adult resistance under field conditions.

## Material and methods

### Plant material

The study was based on 109 doubled haploid lines from a cross between the closely related Norwegian six-rowed barley cultivars Arve (released in 1990, moderately susceptible to NB) and Lavrans (released in 1999, moderately resistant) obtained by microspore culture from F_1_ seeds. The pedigrees of Arve and Lavrans are ‘Otra/Vigdis//Agneta’ and ‘Vera/4/Arve/3/Sold/Alva//Mø75-288’, respectively, with Vera being a sister line of Arve.

### Fungal isolates

Three *P*. *teres* single conidia isolates were used in all experiments in this study. The isolates 5050B and 6949B were isolated from barley seeds collected in Southeastern (5050B) and Northern (6949B) Norway in 2012 and provided by Kimen seed laboratory in Ås, Norway. Isolate LR9 was obtained from barley leaves collected in the Trøndelag area in Norway in 2011. All isolates were confirmed to be NFNB by a polymerase chain reaction (PCR)-based test developed by Williams et al. [18]. The infected plant material was surface sterilized in 70% ethanol for 10 seconds and 0.5% NaOCl for 90 seconds and placed on moist filter paper at 21°C and 12h UV light for approximately 3–5 days until conidia started to develop. Single conidia were transferred to V8 agar plates (150 ml V8 Juice, 10.0 g Difco PDA, 3.0 g CaCO_3,_ 10.0 g agar, 850 ml distilled H_2_O) and after sufficient mycelium development agar plugs with a diameter of 0.6 cm were excised, air-dried and stored at -80°C until further use.

### Field experiments

To produce inoculum for field experiments, each single spore isolate was grown separately from agar plugs on V8 agar plates for 7 days at 20°C in the dark, for 24 hours at 21°C in the light and for 24 hours at 15°C in the dark to promote conidia formation. The plates were flooded with water and the conidia were scraped off the surface with a sterile inoculation loop. For each isolate, the inoculum was diluted to a volume of ca. 3 liters with 1 drop Tween 20 added for every 50 ml of inoculum. The highly susceptible cultivar ‘Tiril’ was grown in trays in the greenhouse at 20–25°C. Each tray was spray-inoculated with one of the three isolates. The inoculation was repeated twice during the course of five weeks to ensure sufficient disease development. After maturation all above ground biomass was harvested, dissected into 5 cm long pieces and the straw inoculated with the different isolates was mixed at equal shares.

The Arve x Lavrans population was sown in hillplots in an alpha lattice design at Vollebekk research farm, Ås, Norway, over three years with two (2014) or three (2015 and 2016) replications. The moderately susceptible cultivar ‘Heder’ was planted at the borders of the field trial to minimize border effects. After approximately one month the plants were inoculated with the infected straw. The field trial was mist-irrigated daily for 10 minutes per hour from 7 to 10 pm in order to promote disease development. In 2015 and 2016 the trial was sprayed with Talius (proquinazid, 40 g/ha) at three-week intervals to control powdery mildew (*Blumeria graminis* f. sp. *hordei*). Disease severity was scored as percentage of infected leaf area based on the whole hillplot at two different timepoints. The first scoring was done when some lines had reached approximately 25% disease severity and the second scoring approximately one week later when they had reached up to 40%. Scoring at early timepoints of disease development was necessary because later in the season accurate scoring would be hampered due to lodging and infection with competing diseases such as powdery mildew or leaf rust (*Puccinia hordei*). In 2014, the population was scored only once due to heavy powdery mildew infection. In addition, days to heading (DH) and plant height (PH) were recorded in all years.

### Greenhouse experiments

For disease phenotyping on seedlings in the greenhouse, the isolates LR9, 5050B and 6949B were grown on V8 agar as described above. The inoculum was diluted to 2000 spores/ml and 1 drop of Tween 20 was added per every 50 ml.

Two seeds per barley line were sown in SC10 plastic cones (Stuewe and Sons, Inc., Corvallis, Oregon, USA) placed into racks of 98 and the plants were grown in the greenhouse at 22°/16°C (day/night), 16 hours light and 65% relative humidity (RH) for two weeks. The susceptible cultivar Tiril was used as a border to minimize border effects and to serve as a control to ensure even inoculation. When the second leaf had fully expanded, the plants were spray-inoculated with the spore suspensions until the leaves were at the point of inoculum runoff. The infected plants were kept in mist chambers at 100% RH, 21°C and continuous light for 24 hours. After 24 hours the plants were moved back to greenhouse chamber conditions. Four to five days after inoculation, the second leaves of both plants from each line were scored together for disease development according to the Tekauz disease reaction type scale where a score of 1 denotes small lesions (resistance) and 10 complete necrosis (susceptibility) [19]. The experiments were performed three times with each isolate.

### Statistical analysis

The PROC GLM procedure in the SAS software package 9.4 (SAS Institute Inc.) was used for Analysis of Variance (ANOVA) analysis. Broad sense heritability within and across years was estimated from the ANOVA table using the formulas h^2^ = σ _g_^2^/(σ _g_^2^+ σ _E_^2^/r) and h^2^ = σ _g_^2^/(σ _g_^2^+ σ_gxy_^2^/y+σ _E_^2^/ry), respectively, with σ _g_^2^ = genetic variance, σ_gxy_^2^ = genotype-by-environment interaction variance, σ _E_^2^ = error variance, r = number of replicates and y = number of years. The LSMEANS function in PROC MIXED was used to calculate the mean NB severity, mean DH and mean PH of each line. To determine whether DH and PH influence the disease development under field conditions, the mean NB severity of every line in every year was regressed to the mean DH and mean PH in the corresponding year using the PROC REG procedure. PH was found to have a significant impact in 2014 and both scorings in 2015 and was used as a covariate in QTL mapping. The Pearson correlation coefficients were calculated with the PROC CORR function.

### Map construction and QTL mapping

Genomic DNA was extracted from young leaves of the parents and all doubled haploid lines using the DNeasy Plant DNA Extraction Kit (Qiagen). The population was genotyped for 7864 markers on the Illumina iSelect 9k Barley SNP Chip (Illumina) at Trait Genetics GmbH (Gatersleben, Germany). SNP markers for which the genotyping failed in more than 10% of the individuals were excluded from further analysis. Out of the remaining 6888 markers, 589 markers were polymorphic and segregated in the population and were used to construct linkage maps. Heterozygous SNPs were treated as missing values. Two lines with more than 10% missing marker data were omitted from further analysis. A genetic linkage map was constructed using the Kosambi function in the software JoinMap 4.0 [20]. Initially, linkage groups were created at an independence LOD (logarithm of odds) score of 3.0. In a second step, the LOD was lowered to 1.9 to obtain separate linkage groups for each chromosome. A recently published consensus map [21] was used to determine which chromosomes the obtained linkage groups represent. Maximum Likelihood mapping was used with default parameters to produce linkage maps.

QTL mapping was performed with the software MapQTL 6 [22]. First, interval mapping (IM) was performed to detect major QTL for NB resistance and then the most closely linked markers to these QTL were used as cofactors for multiple-QTL models (MQM) mapping. In this study, we report on the IM results since MQM did not produce more significant results than IM. The LOD threshold of 2.5 for significance of a QTL was determined by permutation test based on 1000 permutations with α = 0.05 for type 1 error rate. Linkage maps and LOD curves were created with MapChart 2.3 [23]. To allow the comparison of QTL found in this paper with previously described QTL, the marker positions on the consensus map by Muñoz-Amatriaín et al. [24] and on the POPseq map [25] are given wherever appropriate.

### QTL nomenclature

We followed the QTL nomenclature established by Grewal et al. [13], but we did not differentiate between seedling stage and adult stage QTL. A suffix was added to distinguish different QTL on the same chromosome, and the prefix “AL_” was added to designate the name of the population the QTL was found in (Arve x Lavrans).

## Results

### Disease severity

**Fig 1:**
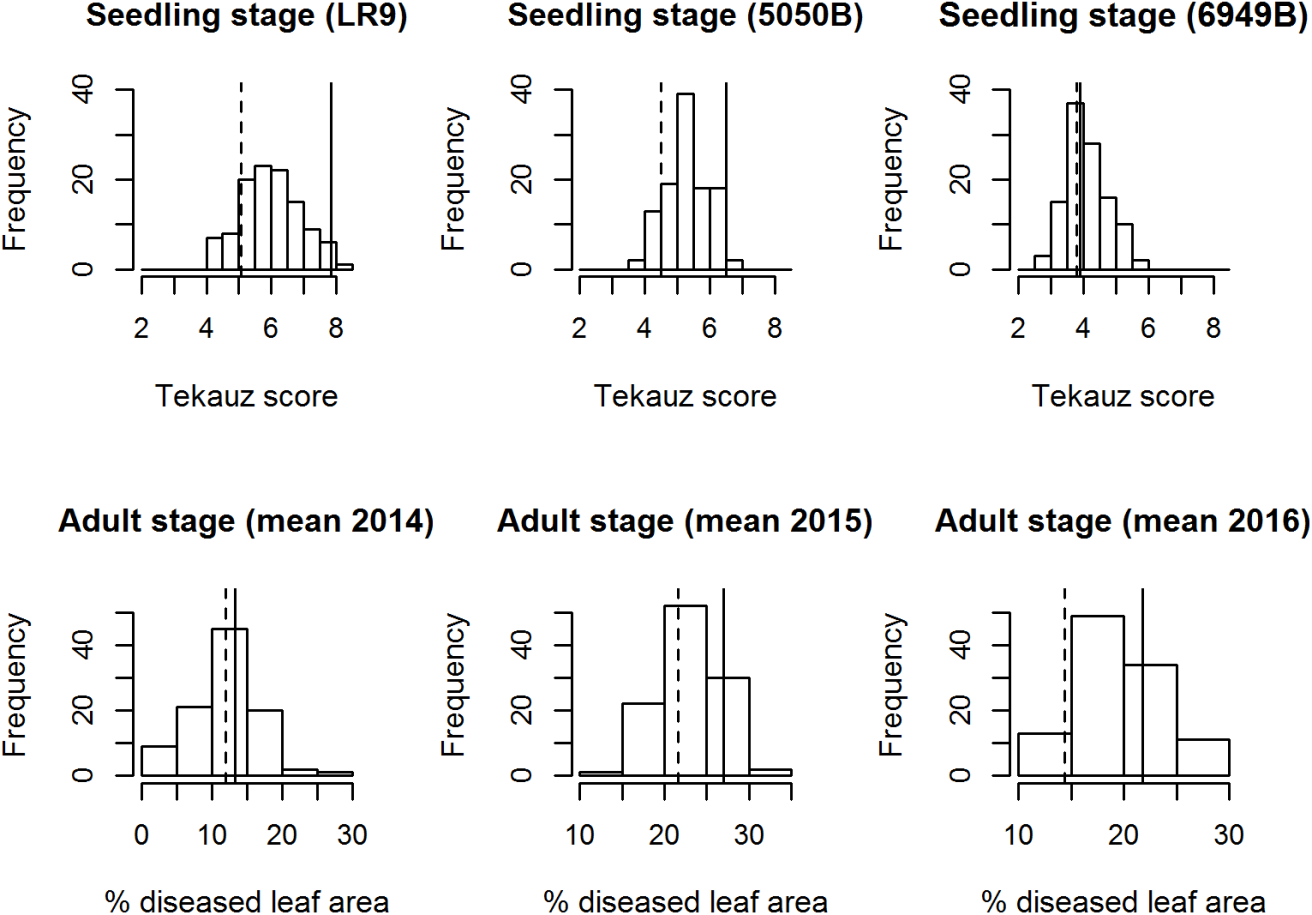
Frequency distributions for disease severities in the Arve x Lavrans mapping population. Disease responses are shown as Tekauz scores in seedling inoculations with three different isolates LR9. 5050B and 6949B and as percentage of diseased leaf area for adult plants under inoculated field conditions in three years. Vertical solid line represents the disease scores of Arve, vertical dashed lines represents disease scores of Lavrans.

Despite the genetic similarity of the parents Arve and Lavrans, the mapping population segregated for NB resistance in seedlings and adult plants, as well as for DH and PH (Fig 1, S1 and S2 Figs). The disease severity followed a normal distribution with transgressive segregation. The adult plant disease scores ranged from 5–30% diseased leaf area in 2014, from 14–33% in 2015 and from 13–30% in 2016, with average disease scores of 14%, 23% and 20%, respectively. On average, the plants were 68 cm in 2014, 94.5 cm in 2015, and 65 cm in 2016, and the average time to heading was 45 days, 66 days, and 45 days, respectively. Seedling inoculations with the LR9 and 5050B isolates yielded Tekauz scale disease scores between 4.2 and 8.2 (average: 6.1), and 4.0 and 7.0 (average: 5.3), respectively, while the 6949B isolate caused symptoms between 3.0 and 6.0 points (average: 4.2) on the scale and thus seems to be slightly less aggressive than the other two isolates. Whereas the isolates LR9 and 5050B produced typical NFNB net-shaped symptoms ca. 4–5 days after inoculation, we observed that the symptoms caused by 6949B remained smaller and spot-shaped before expanding into the typical net symptoms at ca. 6–7 days after inoculation. Consistent with previous characterization of Arve as being more susceptible to NB than Lavrans, Arve reached higher disease scores in all environments. These differences were significant during infection with LR9 and 5050B at the seedling stage and in adult plants in 2016 (Fig 1). No significant differences in DH and PH were observed between the parental lines.

Significant correlations of adult plant disease severities were observed between all years (Table 1). The correlations between 2014 and 2016 and 2015 and 2016 were 0.41 and 0.48, respectively (p<0.001), while the correlation between 2014 and 2015 was slightly lower (r = 0.23, p<0.05). In the 2014 field trial, the NB severity was significantly correlated with PH (r = 0.31) but not with DH (r = 0.06). However, a significant correlation to the heading dates scored in 2015 and 2016 was found (0.33 and 0.29, respectively). The NB scores in 2015 were significantly correlated with PH (r = 0.25, p<0.05), but not with DH (r = -0.10). Conversely, NB scores in 2016 were significantly correlated with DH (0.19, p<0.05) but not with PH (-0.10). The correlation between resistance and PH was negative in that year. The correlation between seedling experiments and adult plant field trials ranged between r = 0.31 and r = 0.59 and was highly significant in all cases. Notably, there was also a significant correlation between seedling inoculations and DH in 2015 and 2016 as well as PH in 2015. Heritability of disease severity across years was 0.69 and that of DH and PH 0.79 and 0.73, respectively (Table 2). The heritability of resistance within year ranged from 0.77 to 0.89 under field conditions and from 0.90 to 0.95 in seedling inoculations (Table 1). Analysis of variance showed significant differences (p<0.0001) among genotypes and years for resistance.

**Table 1:** Pearson correlation coefficients for net blotch severities, DH and PH and heritability (h^2^) within years.

	NFNB severities in adult plants							NFNB severities in seedling tests			Days to heading (DH)			Plant height (PH)		
	2014	2015_1^a^	2015_2	2015	2016_1	2016_2	2016	LR9	5050B	6949B	2014	2015	2016	2014	2015	2016
NB2015_1	0.27**															
NB2015_2	0.17	0.58***														
NB2015	0.23*	0.79***	0.93***													
NB2016_1	0.43***	0.51***	0.44***	0.48***												
NB2016_2	0.39***	0.50***	0.40***	0.45***	0.78***											
NB2016	0.41***	0.53***	0.43***	0.48***	0.87***	0.99***										
LR9	0.39***	0.48***	0.32***	0.37***	0.49***	0.44***	0.47***									
5050B	0.46***	0.48***	0.37***	0.44***	0.43***	0.46***	0.47***	0.68***								
6949B	0.39***	0.35***	0.29**	0.31**	0.56***	0.56***	0.59***	0.52***	0.55***							
DH2014	0.06	0.00	-0.09	-0.08	0.07	0.15	0.14	0.21*	0.11	0.26						
DH2015	0.33**	-0.05	-0.09	-0.10	0.20*	0.17	0.19	0.28**	0.31**	0.28**	0.58***					
DH2016	0.29**	0.05	0.05	0.02	0.21*	0.18	0.19*	0.34***	0.35***	0.41***	0.64***	0.77***				
PH2014	0.31**	-0.06	0.02	-0.05	0.04	0.04	0.05	0.14	0.11	0.00	0.11	0.21*	0.12			
PH2015	0.28**	0.27**	0.22*	0.25*	0–09	0.12	0.12	0.24*	0.23*	0.14	0.13	0.33***	0.28**	0.42***		
PH2016	0.18	0.03	-0.06	-0.08	-0.06	-0.11	-0.10	0.10	0.05	0.08	0.12	0.34***	0.27	0.48***	0.68***	
h^2^	0.77	0.80	0.80	0.80	0.87	0.89	0.89	0.91	0.90	0.95	0.74	0.95	0.91	0.73	0.90	0.92

* <0.05.

** <0.01.

*** <0.001

^a^ Net blotch scores: Numbers before and after the underscore in the trait name represent the year and number of scoring, respectively

**Table 2:** Analysis of variance table for net blotch severity (NB), days to heading (DH) and plant height (PH) and heritabilities in the AxL mapping population.

Trait	Source	df	Mean square	F value	P value	Heritability
NB^a^	Genotype	107	50.00	2.73	<0.0001	0.69
	Year	1	444.38	24.23	<0.0001	
	Genotype x year	102	18.34	1.39	0.0230	
	Rep(Year)	4	36.13	2.73	0.0299	
	Block(Rep x year)	54	19.60	1.48	0.0256	
	Error	228	13.23			
DH^b^	Genotype	108	5.58	5.57	<0.0001	0.79
	Year	3	27029.75	26974.99	<0.0001	
	Genotype x year	205	1.00	1.88	<0.0001	
	Rep(Year)	5	2.56	4.30	0.0008	
	Block(Rep x year)	67	0.77	1.29	0.0707	
	Error	420	0.60			
PH^c^	Genotype	108	70.81	4.09	<0.0001	0.73
	Year	3	1063755.86	61396.51	<0.0001	
	Genotype x year	205	17.33	1.42	0.0019	
	Rep(Year)	5	90.00	7.37	<0.0001	
	Block(Rep x year)	67	16.20	1.33	0.0555	
	Error	370	12.21			

^a^ NB: net blotch severity

^b^ DH: days to heading

^c^ PH: plant height

### Map construction

Two doubled haploid lines with more than 10% missing marker data were omitted from further analysis. A total of 589 SNP markers was polymorphic in the population and was used to construct a linkage map which spanned 644.9 cM in total (Table 3). Due to the close relatedness of the parental lines, no segregating markers were found on chromosome 1H, and chromosomes 2H, 4H, 5H and 6H contained major gaps of 58.5 cM, 33.3 cM, 43.3 cM and 50.5 cM, respectively. Two linkage groups were obtained for chromosome 3H and 5H. Marker density ranged from 0.4 cM (3H.1) to 2.9 cM between markers (linkage group 5H.2 on chromosome 5H). The marker positions in the Arve x Lavrans map were found to be in good agreement with the recently published consensus map [21] (see S1 Table for a comparison of the marker positions on both maps).

**Table 3:** SNP coverage and distribution across all chromosomes after filtering.

Linkage group	cM	Markers	Marker coverage (cM/marker)
1H	-	-	-
2H	108.1	71	1.5
3H.1	23.4	57	0.4
3H.2	2.9	5	0.6
4H	156.2	139	1.1
5H.1	98.1	65	1.5
5H.2	31.7	11	2.9
6H	142.4	87	1.6
7H	82.1	154	0.5

### QTL mapping

**Fig 2:**
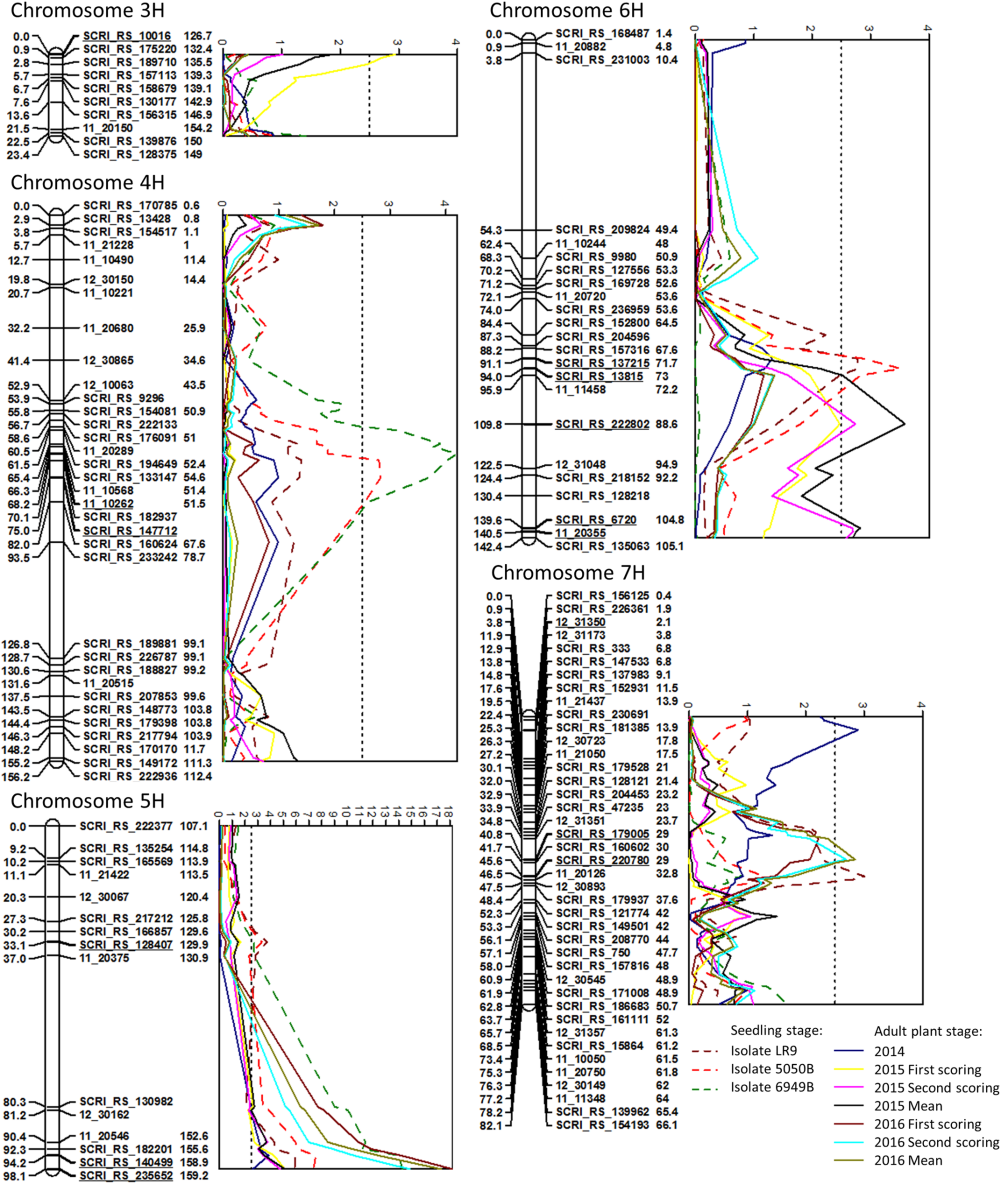
Chromosomes with significant QTL for net blotch resistance with LOD curves obtained with interval mapping. Genetic distances on the AxL map are given in cM on the left side of the linkage map bars. The numbers on the right side of the marker names refer to the POPseq position of the marker [25]. Only one marker per position was kept. Markers most closely linked to QTL are underlined. The dashed lines indicate the LOD threshold of 2.5 determined by permutation test.

In total, nine QTL significantly associated with resistance to NB were found on chromosomes 3H, 4H, 5H, 6H and 7H in different years and at different developmental stages using interval mapping (Fig 2 and Table 4). Chromosome 6H harbored three QTL, while chromosomes 5H and 7H contained two QTL and 3H and 4H one QTL each. In adult plants assessed under field conditions, 1–3 QTL were detected in the different scorings. Four QTL were found in seedling inoculations with LR9 and 5050B, while three QTL were detected in inoculations with 6949B. A major QTL on chromosome 5H (AL_QRptt5-2) peaking around 98.1 cM between the markers SCRI_RS_140499 and SCRI_RS_8410 was consistently found under inoculations with all three isolates at the seedling stage and in all field trials, explaining between 15.5% (2014) and 54.7% (first scoring 2016) of the genetic variation. Apart from AL_QRptt5-2, only one QTL was significantly associated with resistance in both seedlings and adult plants. AL_QRptt7-2 at 41–46 cM on 7H was detected in seedling inoculations with LR9 and in adult plants in 2016, explaining around 12% of the genetic variation. Four QTL were only significant in adult plants. AL_QRptt3-1 (0 cM) was significant only in the first scoring in 2015 and explained up to 10.8% of the genetic variation while AL_QRptt7-1 (3.8 cM) explained up to 11.9% in 2014. However, we cannot exclude the possibility that AL_QRptt7-1 was caused by faulty scoring in 2014 due to heavy infection with powdery mildew. The two adult stage QTL on 6H, AL_QRptt6-2 (110 cM) and AL_QRptt6-3 (~140 cM), were only significant in 2015 and explained up to 14.8% and 11.0%, respectively. Three QTL were only detectable at the seedling stage. AL_QRptt5-1 peaked at 33 cM on 5H and was significant during inoculations with all three isolates. It explained between 11.5% and 14.8% of the genetic variation in these experiments. AL_QRptt4-1 close to the markers SCRI_RS_147712 and 11_10262 at 68.2–75.0 cM on 4H was significant under inoculations with 5050B and 6949B and explained up to 16.5% of the genetic variation. A QTL on 6H (AL_QRptt6-1) peaked at 94.0 cM in the vicinity of the marker SCRI_RS_13815. It explained up to 14.0% of the genetic variation and was detected in seedling resistance assessment with LR9 and 5050B. This QTL was not found during inoculation with 6949B, indicating a race-specific resistance mechanism at this locus. Except for AL_QRptt4-1, the resistance is conferred by the more resistant parent Lavrans at all loci.

**Table 4:** QTL for net blotch severity in the Arve x Lavrans mapping population.

	AL_QRPtt3-1			AL_QRptt4-1			AL_QRptt5-1			AL_QRptt5-2			AL_QRptt6-1			AL_QRptt6-2			AL_Qrptt6-3			AL_QRptt7-1			AL_QRptt7-2		
Closest marker	SCRI_RS_10016			SCRI_RS_147712 (5050B), 11_10262 (6949B)			SCRI_RS_128407			SCRI_RS_140499 (NB14, LR9, 5050B), SCRI_RS_235652			SCRI_RS_137215 (LR9), SCRI_RS_13815 (5050B)			SCRI_RS_222802			11_20355 (NB15_2), SCRI_RS_6720 (NB15)			12_31350			SCRI_RS_179005 NB16_2, NB16), SCRI_RS_220780 (LR9)		
AxL map position (cM)	0.0			75.0, 68.2			33.1			94.2, 98.1			91.1, 94.0			109.8			140.5, 139.6			3.8			40.7, 45.6		
Consensus map range of the most significant markers [24]	128.5–129.6			58.7–59.2; 55.0			128.8–130.4			NA; 170.0–170.1			77.7–78.1; 79.8–80.3			94.5–96.2			113.1; 112.8			3.21			NA; NA		
POPseq map range of the most significant markers [25]	126.0–128.6			57.5–59.7; 51.4			129.9–130.7			158.9; 159.2–159.8			71.7, 73.0			87.6–88.6			NA, 104.8			2.0			26.7–31.4; 29,0		
Trait	LOD	Add ^a^	R^2^ (%) ^b^	LOD	Add	R^2^ (%)	LOD	Add	R^2^ (%)	LOD	Add	R^2^ (%)	LOD	Add	R^2^ (%)	LOD	Add	R^2^ (%)	LOD	Add	R^2^ (%)	LOD	Add	R^2^ (%)	LOD	Add	R^2^ (%)
NB14										3.8	2.0	15.5										2.9	1.7	11.9			
NB15_1^d^	2.9	1.2	10.8							5.1	1.4	18.0				2.6	1.2	9.7									
NB15_2										4.8	2.0	18.1				2.8	1.6	10.9	2.7	1.5	10.7						
NB15										5.1	1.6	19.0				3.7	1.5	14.8	2.8	1.2	11.0						
NB16_1										18.2	1.9	54.7															
NB16_2										14.9	5.4	47.6													2.7	2.7	11.1
NB16										17.3	3.7	53.1													2.9	1.8	11.8
LR9							3.7	0.3	14.8	6.2	0.5	23.4	2.9	0.3	11.6										3.0	0.4	12.1
5050B				3.0	-0.2	12.0	3.3	0.2	13.4	7.8	0.4	28.6	3.5	0.3	14.0												
6949B				4.2	-0.3	16.5	2.8	0.2	11.5	15.0	0.5	47.6															
Res. source	L^c^			A			L			L			L			L			L			L			L		

^a^ Additive effect.

^b^ Percent of phenotypic variance explained by QTL.

^c^ A: Arve. L: Lavrans.

^d^ Net blotch scores: Numbers before and after the underscore in the trait name represent the year and number of scoring, respectively.

**Fig 3:**
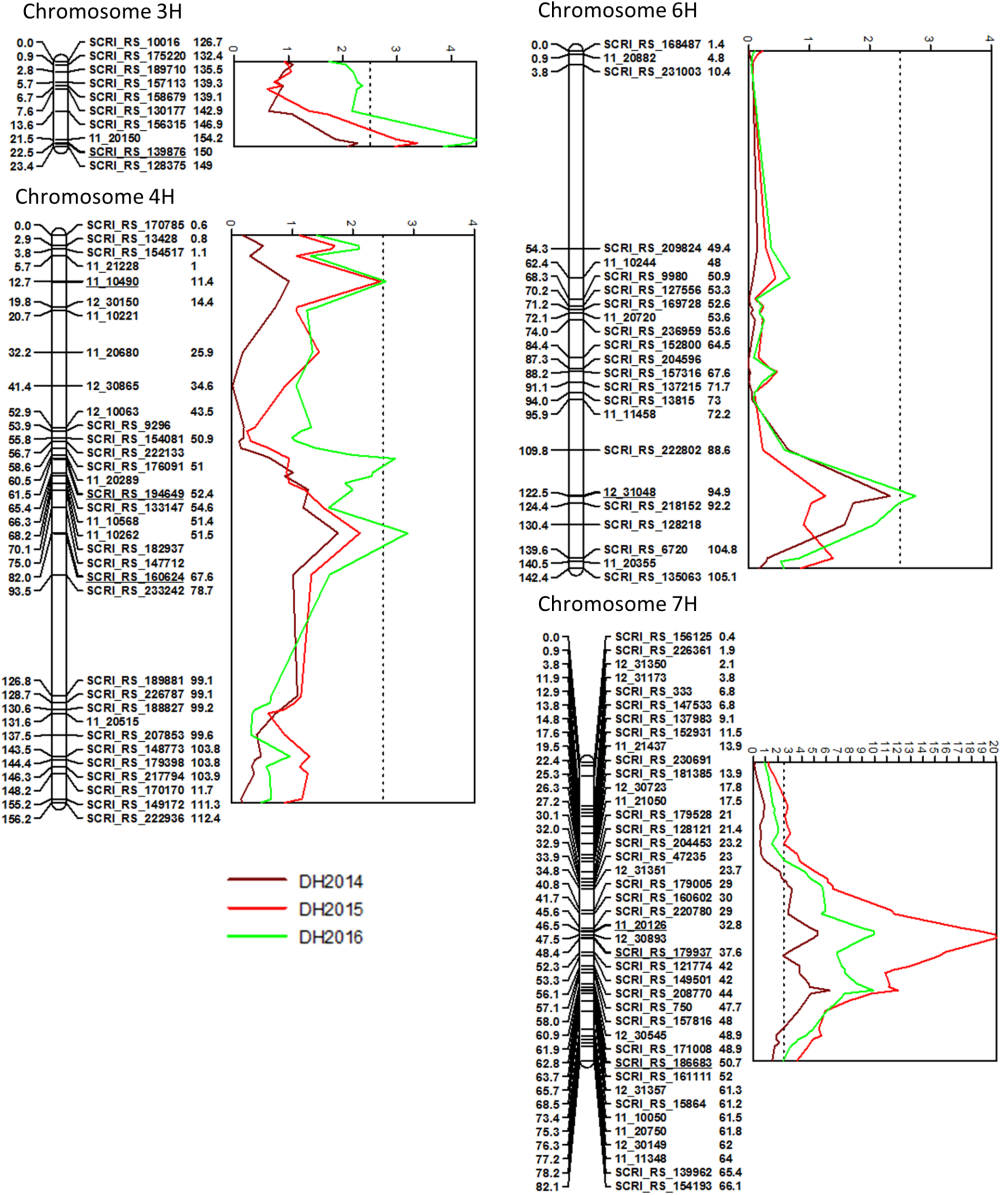
Chromosomes with significant QTL for DH in the Arve x Lavrans population with LOD curves obtained with interval mapping. Genetic distances on the AxL map are given in cM on the left side of the linkage map bars. The numbers on the right side of the marker names refer to the POPseq position of the marker [25]. Only one marker per position was kept. The dashed lines indicate the LOD threshold of 2.5 determined by permutation test.

In total, five QTL for DH were found on chromosomes 3H, 4H and 6H (Fig 3, S2 Table). On chromosome 7H, significant marker-trait associations (MTA) were found within an interval from 12 to 82 cM. The most significant markers were located at 46.5–48.4 cM and 62.8 cM on chromosome 7H and were significant in all three years and explained up to 57.8% and 40.2% of the genetic variation, respectively. A QTL at 22.5 cM on 3H was significant in 2015 and 2016 (13.5% and 17.2%). Chromosome 4H harbored three regions at 12.8 cM, 61.4 cM and 82.0 cM which were significantly associated with DH in 2016, explaining between 10.4% and 11.8%. Additionally, 11.3% of the variation were explained by a QTL at 122.5 cM on 6H.

**Fig 4:**
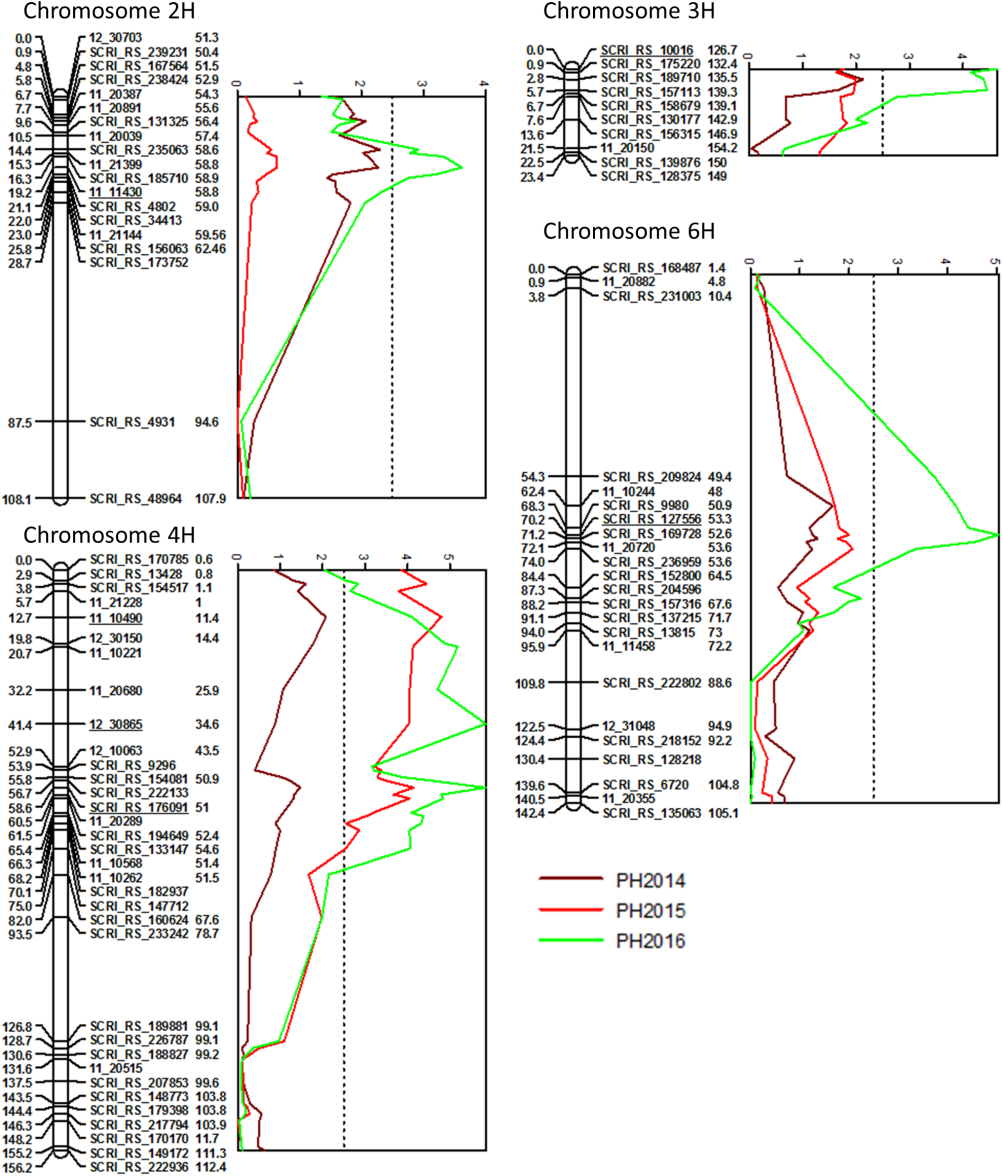
Chromosomes with significant QTL for PH in the Arve x Lavrans population with LOD curves obtained with interval mapping. Genetic distances on the AxL map are given in cM on the left side of the linkage map bars. The numbers on the right side of the marker names refer to the POPseq position of the marker [25]. Only one marker per position was kept. The dashed lines indicate the LOD threshold of 2.5 determined by permutation test.

Four PH QTL were found on 2H, 3H, 4H and 6H (Fig 4, S3 Table). The region significantly associated with PH on chromosome 4H spanned a region of 75 cM and it is not clear how many QTL are present. The LOD curve showed a peak at 58.6 cM in 2015 and 2016, explaining up to 22.4% of the genetic variation. In addition, two more peaks were at 12.7 cM and 41.4 cM on the same chromosome in 2015 and 2016, respectively, but it remains to be elucidated if these peaks represent separate QTL. In 2016, three additional QTL were located at 19.2 cM on 2H, 0.0 cM on 3H and 70.2 cM on 6H, explaining 14.5%, 18.3% and 19.7%, respectively. Most of these QTL were also observed in 2014, but did not reach the significance threshold.

The PH QTL on 3H and 4H co-located with the NB resistance QTL AL_QRptt3-1 and AL_QRptt4-1 and the 7H QTL for DH partly overlapped with AL_QRptt7-2. The AL_QRptt4-1 region coincided with markers significantly associated with DH in 2016 and PH in 2015 and 2016.

## Discussion

### Disease severity

In both seedlings and adult plants, quantitative variation in disease severity was observed in the Arve x Lavrans population, suggesting the involvement of multiple genes in NB resistance, which is confirmed by the results of the QTL analysis.

The field trials were inoculated with the same isolates used for seedling inoculations in the greenhouse. In spite of the likely presence of natural inoculum in the field, the different developmental stages of the plants tested and the different scales used to score the disease, a significant correlation of disease severity was observed between the two sets of experiments (r = 0.31–0.59). Even though the natural *P*. *teres* population in the field is likely to differ between years, this correlation was relatively stable across years, indicating that most of the observed disease symptoms were caused by the three isolates or genetically similar naturally occurring isolates. Seven of the 20 lines that were most resistant at the seedling stage were also among the 20 most resistant lines under field conditions. Similarly, 10 of the 20 most susceptible lines under greenhouse conditions were also among the 20 most susceptible lines in the field. However, among these 20 most resistant or susceptible lines under each condition were usually one or two lines that ranged in the opposite group under the respective other condition. Thus, a line that is resistant at the seedling stage may still show substantial susceptibility under field conditions. These observations indicate that it is possible to use seedling screenings with isolates representative of the natural pathogen population to pre-screen breeding material before field testing, under the condition that the selection criteria are not too strict. In a similar study, Grewal et al. reported a higher correlation (r = 0.65–0.71) between seedling inoculations with different isolates and adult plant-stage reactions in non-inoculated field experiments than in our study [13]. The reason for this difference could be that the isolates used in Grewal’s study are more representative for the natural NB population present at the field site. Another reason could be that the disease scale the authors used for scoring the field trial correlated better with the scale used in seedling experiments than in our study.

### QTL mapping

Despite the close relatedness of the parents, we were able to identify nine NB resistance QTL in this study, demonstrating that QTL mapping can be a powerful tool even in populations derived from narrow crosses. One QTL was highly significant in all scorings, five QTL occurred in more than one experiment and three QTL were present under one condition each. The putatively novel QTL AL_QRptt5-2 at 98.1 cM on 5H (Consensus map: 170.0–170.1 cM, POPseq: 158.9–159.8 cM [25]) was found in all years of field experiments with adult plants and seedling inoculations with three different isolates. This QTL explained a considerable part of the genetic variance, and its stability throughout different environments suggests that the gene underlying this QTL confers broad-range resistance in various environments, which makes it a promising candidate for implementation in resistance breeding. For this, further investigation of this locus, e.g. by fine-mapping, and the identification of closely linked markers is needed. AL_QRptt7-2 was also associated with resistance in both seedlings and adults. These findings indicate that a part of the NB resistance in seedlings and adult plants is conferred by the same genes. AL_QRptt4-1, AL_QRptt5-1 and AL_QRptt6-1 were found in seedling inoculations with at least two isolates. They were not significant in field experiments, but the LOD curves for these traits suggest that these loci still might have a small effect on resistance under field conditions (Fig 2). The other QTL AL_QRptt3-1, AL_QRptt6-2, AL_QRptt6-3 and AL_QRptt7-1 were only significant in one environment each in field trials, so they might represent resistance against naturally occurring NB strains. Further tests under different environments or with additional isolates will be required in order to test this hypothesis and to determine how stable these QTL are.

A number of QTL found in this study have already been described in the literature, while others are putatively novel. Tamang et al. identified markers at 53.7–59.2 cM (consensus map) on chromosome 4H associated with seedling resistance against the two SFNB isolates NZKF2 and DEN.2 from New Zealand and Denmark, respectively, and this region co-locates with AL_QRptt4-1 found in this study [26]. One of the markers significantly associated with resistance to NZKF2 and DEN2.6 is also present in the AxL linkage map and showed significant association with seedling resistance to 6949B. Afanasenko et al. found seedling resistance against a Russian NB isolate in this region (marker 11_11207 at 56.7 cM on the consensus map) [27]. In an association mapping study of 1050 globally collected barley accessions, Richards et al. identified a marker-trait association at 52.7 cM on the POPseq map with seedling resistance to the isolates 6A and LDN [28]. This marker is within the AL_QRptt4-1 region. Additionally, the QRpts4 locus described by Grewal et al. conferring seedling resistance against both NFNB and SFNB isolates is located in close vicinity [13]. These results suggest that this locus is an important seedling resistance QTL which is effective against a number of both NFNB and SFNB isolates from different regions of the world. It is not clear yet, however, if the QTL presented in these studies are identical or if this region harbors multiple resistance genes.

The QTL AL_Qrptt3-1 and AL_QRptt5-1 both co-locate with two resistance QTL described by Afanasenko et al. against the Russian isolates PL9 and PP7, respectively [27]. AL_QRptt3-1, which is at 128.5–129.6 cM on the POPseq map, is in close vicinity of the marker 11_20920 at 123.23 cM, and the marker 11_10845 at 129.44 cM on the POPseq map is within the AL-QRptt5-1 interval of 129.9–130.7 cM [27]. In addition, Afanasenko et al. found an association of the marker 11_20531 (POPseq map: 94.9 cM) with resistance to the two Russian net form isolates PP1 and PP6. This marker is located between the two QTL AL_QRptt6-2 (POPseq: 87.6–88.6 cM) and AL_QRptt6-3 (POPseq: 104.8 cM), and it remains to be elucidated whether this marker represents a separate QTL or one of the two QTL.

Due to the close relatedness of Arve and Lavrans, many of the genotyped markers were not polymorphic in the population, resulting in several gaps in the map. The LOD curve for AL_QRptt5-2 increased until the end of the map, suggesting that the true location of the QTL might be beyond the map. It will thus be useful to map this QTL in other populations in order to determine its precise location.

### Potential effect of PH and DH on NB resistance

It is important to distinguish between ‘true’ disease severity and the possible confounding influences of other developmental factors such as PH and DH. It is conceivable that the top leaves of tall plants might remain healthy for a longer period than those of shorter plants because they can escape the fungus more easily. Lines with an early heading date develop faster and might be exposed to the pathogen for a longer time than late lines. Early maturation of plants may promote disease development of necrotrophic pathogens due to facilitated infection of senescing leaves. Additionally, in maturing plants accurate disease severity assessment may be hampered by the confusion of infected leaf tissue with naturally senescing tissue. In this study, we found that PH had a significant (P<0.05) effect on disease severity in two years and was thus used as a covariate in QTL mapping. Interestingly, the correlation in 2014 and 2015 was significant and positive, indicating that taller plants were more susceptible than shorter plants. In 2016, taller plants tended to be more resistant than shorter ones, although this association was not significant. A significant positive correlation was also found between PH and disease severity after seedling inoculations with two of three isolates. Since we can rule out an effect of PH on disease severity in two-week old spray-inoculated seedlings, we assume that this correlation is not caused by plant architecture but rather is of genetic nature, i.e. determined by closely linked genes or by one gene with a pleiotropic effect on both traits. Two of the loci associated with NB resistance, AL_QRptt3-1 and AL_QRptt4-1, were significantly associated with PH in this study. At both loci, low plant height was conferred by the allele from Arve, while the allele conferring resistance was from Arve on AL_QRptt3-1, and from Lavrans on AL_QRptt4-1. In an association mapping study identifying QTL for a number of agronomic traits in a global collection of spring barley, Pasam et al. identified the PH QTL QTL16_PHT associated with PH at 58.9 cM (consensus map) on 6H which co-locates with one of the PH QTL found in this study. Additionally, the authors identified the PH QTL QTL3_PHT on 4H at 69 cM on the consensus map, and this region was also significantly associated with PH in this study. Pasam et al identified this QTL as a QTL previously detected in a Harrington x Morex cross by Marquez-Cedillo et al. [29]. Previous studies reported that dwarfing and semi-dwarfing genes determining PH in barley are able to confer increased disease resistance against necrotrophic pathogens through attenuation of phytohormone pathways such as the brassinosteroid (BR) or salicylic acid and jasmonic acid pathways [30, 31]. Loci conferring resistance against other barley diseases such as Fusarium crown rot and Fusarium Head Blight have also been reported to co-locate with PH loci [32–34]. In the case of the AL_QRptt3-1 and AL_QRptt4-1 loci, however, further studies are required to investigate the interactions of resistance and plant height.

In this study, we could not find a clear association between NB resistance and DH. While there was a weak positive correlation between these traits in 2014, it was weakly negative in 2015. In 2016, the correlation was positive and significant. This might be due to different sowing times and different weather conditions during early plant development in the different years. Conversely, we found highly significant positive correlations between DH in two years and NB resistance in seedlings (p<0.001), which indicates either genetic linkage or pleiotropy. Pasam et al. found the DH QTL QTL16_HD on chromosome 7H, which the authors identified as the previously described HvCO1 locus [35, 36]. This locus co-locates with the QTL we identified at 46.5–48.4 cM (consensus map: 38.3–43.4 cM) [37]. HvCO1 is a regulator of flowering induction in response to photoperiod [38]. The HvCO1 locus did not have any effect on other agronomic traits [35], and it is not known if it influences disease resistance. The resistance QTL AL_QRptt4-1 and AL_QRptt7-2 were significantly associated with DH. To date, the effect of earliness on net blotch resistance has not been established. Spaner et al. identified a multi-disease resistance locus in TR306 x Harrington against net blotch, stem rust and scald in a region on chromosome 4H which is associated with DH and the authors speculate that different maturity times may constitute a disease escape mechanism, but this has not been established yet [39, 40]. In FHB infected plants, resistance is usually associated with a late heading date, and regions on chromosomes have been associated with both FHB resistance and heading date [41, 42].

## Conclusions

In this study, nine QTL associated with NB resistance were found on all chromosomes except 1H and 2H in the Arve x Lavrans mapping population, suggesting that the disease is controlled by several genes, most of them with relatively moderate effects. The most significant QTL AL_QRptt5-2 was observed in all environments and developmental stages and explained up to 54.7% of the genetic variance, making it a very promising candidate for introducing stable NB resistance into barley breeding programs. Eight other QTL on 3H, 4H, 5H, 6H and 7H were present in at least one of the conditions tested. The QTL that were only found in one environment are likely to represent defense mechanisms that are only functional in certain environments or against a small number of isolates or may be attributed to naturally occurring NB isolates in the field. AL_QRPtt6-1 was race-specific and was not found in inoculations with 6949B.

Inoculations with more isolates from different regions worldwide and under different environmental conditions will clarify whether these QTL represent general or race-specific defense mechanisms. Further work will include the validation of the QTL in other populations. Combining resistance genes functional in all growth stages and against a range of isolates will be most effective in breeding for stable resistance to net blotch in Norwegian barley cultivars. Understanding the molecular background of the barley NB pathosystem will allow for more efficient resistance breeding of locally adapted cultivars that maintain yield and quality under the current climatic and environmental conditions and thus contribute to a sustainable and integrated approach to disease management.

Since the genetic map of the Arve x Lavrans population contains major gaps, additional QTL can be expected to be present in this population. All but one resistance QTL found in this study were contributed by the moderately resistant parent Lavrans, making this variety a promising candidate for further investigation.

## Supporting information

S1 Fig Frequency distributions for disease severities in adult plants in both scorings in 2015 and 2016. Vertical solid line represents the disease scores of Arve, vertical dashed lines represents disease scores of Lavrans.
(TIF)

S2 Fig Frequency distributions for DH (top) and PH (bottom) in the Arve x Lavrans mapping population in three years. Vertical solid line represents the disease scores of Arve, vertical dashed lines represents disease scores of Lavrans.
(TIFF)

S1 Table Correlation of marker positions between consensus map and AxL map. Included are markers mapped in both maps.
(XLSX)

S2 Table QTL for earliness (DH) in the Arve x Lavrans mapping population. (DOCX)

S3 Table QTL for plant height (PH) in the Arve x Lavrans mapping population. (DOCX)
