# Supplementary figures and images for: Mapping of quantitative trait loci associated with resistance to net form net blotch (Pyrenophora teres f. teres) in a doubled haploid Norwegian barley population
Source: PLoS One. 2017 Apr 27;12(4):e0175773. doi: 10.1371/journal.pone.0175773 (PMC5407769; doi:10.1371/journal.pone.0175773)

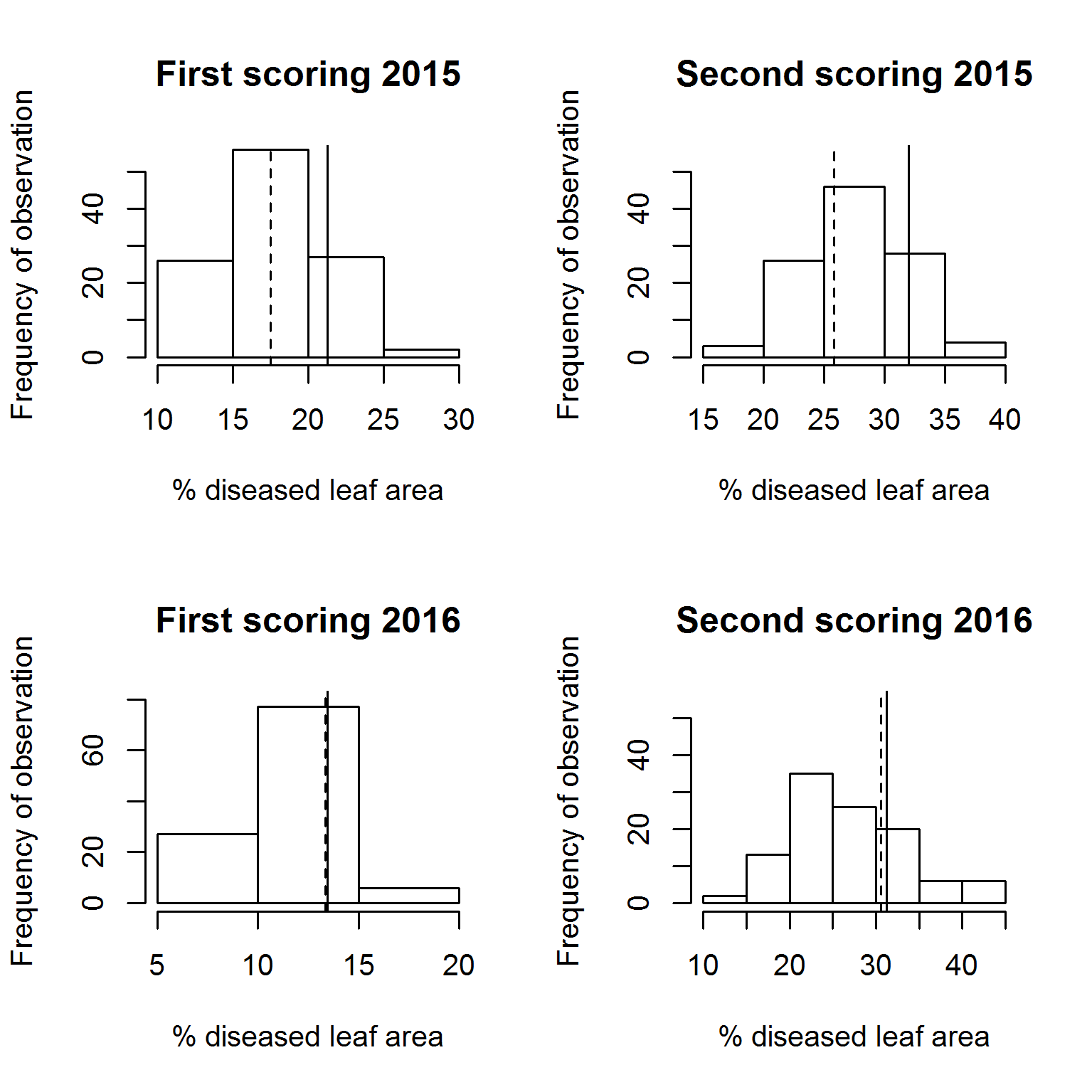

Supplement: S1 Fig — Vertical solid line represents the disease scores of Arve, vertical dashed lines represents disease scores of Lavrans. (TIF) [file pone.0175773.s001.tif]

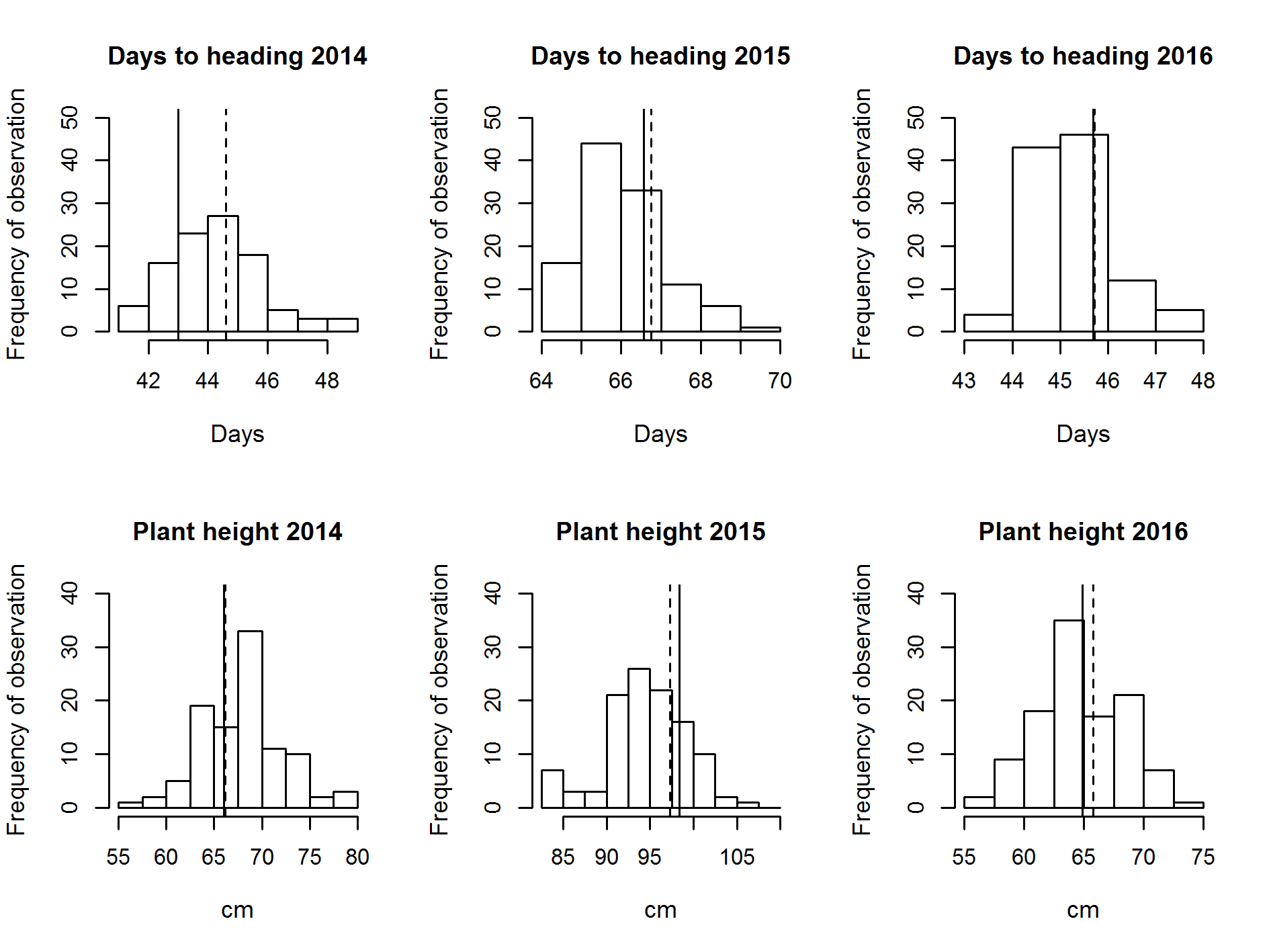

Supplement: S2 Fig — Vertical solid line represents the disease scores of Arve, vertical dashed lines represents disease scores of Lavrans. (TIFF) [file pone.0175773.s002.tiff]
