# Supplementary material for: Mapping of quantitative trait loci associated with resistance to net form net blotch (Pyrenophora teres f. teres) in a doubled haploid Norwegian barley population
Source: PLoS One. 2017 Apr 27;12(4):e0175773. doi: 10.1371/journal.pone.0175773 (PMC5407769; doi:10.1371/journal.pone.0175773)
Supplement: S2 Table — (DOCX) [file pone.0175773.s004.docx]

**S2 Table** QTL for earliness (DH) in the Arve x Lavrans mapping population.

| Chromosome | 3H | | | 4H | | | 4H | | | 4H | | | 6H | | | 7H | | | 7H | | |
| --- | --- | --- | --- | --- | --- | --- | --- | --- | --- | --- | --- | --- | --- | --- | --- | --- | --- | --- | --- | --- | --- |
| Closest marker | SCRI_RS_139876 | | | 11_10490 | | | SCRI_RS_194649 | | | SCRI_RS_160624 | | | 12_31048 | | | 11_20126  (DH14, DH16), SCRI_RS_179937 (DH15) | | | SCRI_RS_ 186683 | | |
| AxL map position (cM) | 22.5 | | | 12.8 | | | 61.4 | | | 82.0 | | | 122.5 | | | 46.5, 48.4 | | | 62.8 | | |
| Consensus map range of the most significant markers [24] | 160.0-164.4 | | | 14.0 | | | 58.9 | | | 67.1-67.6 | | | 102.0 | | | 38.3;43.1-43.4 | | | 58.0-58.9 | | |
| POPseq position  [25] | 150.0-154.9 | | | 11.4 | | | 52.3-53.5 | | | 67.1-67.6 | | | 94.9 | | | 32.8, 37.6 | | | 48.9-50.7 | | |
| Trait | LOD | Add ^a^ | R^2^ (%) ^b^ | LOD | Add | R^2^ (%) | LOD | Add | R^2^ (%) | LOD | Add | R^2^ (%) | LOD | Add | R^2^ (%) | LOD | Add | R^2^ (%) | LOD | Add | R^2^ (%) |
| DH14 |  |  |  |  |  |  |  |  |  |  |  |  |  |  |  | 5.3 | 0.86 | 22.3 | 6.3 | 0.9 | 25.9 |
| DH15 | 3.4 | -0.4 | 13.5 |  |  |  |  |  |  |  |  |  |  |  |  | 20.2 | 0.95 | 57.8 | 11.9 | 0.8 | 40.2 |
| DH16 | 4.3 | -0.4 | 17.2 | 2.5 | -0.27 | 10.4 | 2.7 | -0.28 | 11.0 | 2.9 | -0.29 | 11.8 | 2.7 | -0.29 | 11.3 | 10.0 | 0.59 | 35.1 | 9.9 | 0.6 | 35.0 |
| Allele conferring earliness | A ^c^ | | | A | | | A | | | A | | | A | | | L | | | L | | |

^a^ Additive effect. ^b^ Percent of phenotypic variance explained by QTL. ^c^ A: Arve. L: Lavrans.
