# Supplementary material for: Mapping of quantitative trait loci associated with resistance to net form net blotch (Pyrenophora teres f. teres) in a doubled haploid Norwegian barley population
Source: PLoS One. 2017 Apr 27;12(4):e0175773. doi: 10.1371/journal.pone.0175773 (PMC5407769; doi:10.1371/journal.pone.0175773)
Supplement: S3 Table — (DOCX) [file pone.0175773.s005.docx]

**S3 Table** QTL for plant height (PH) in the Arve x Lavrans mapping population.

| Chromosome | 2H | | | 3H | | | 4H | | | 4H | | | 4H | | | 6H | | |
| --- | --- | --- | --- | --- | --- | --- | --- | --- | --- | --- | --- | --- | --- | --- | --- | --- | --- | --- |
| Closest marker | 11_11430 | | | SCRI_RS_10016 | | | 11_10490 | | | 12_30865 | | | SCRI_RS_176091 | | | SCRI_RS_127556 | | |
| AxL map position (cM) | 19.2 | | | 0.0 | | | 12.7 | | | 41.4 | | | 58.6 | | | 70.2 | | |
| Consensus map range of the most significant markers [24] | 69.6 | | | 128.5-129.6 | | | 14.0 | | | 38.4 | | | 53.7-53.9 | | | 57.9-58.3 | | |
| POPseq position [25] | 58.8 | | | 126.0-128.6 | | | 11.4 | | | 34.6 | | | 51.0-51.1 | | | 52.6-53.3 | | |
| Trait | LOD | Add ^a^ | R^2^ (%) ^b^ | LOD | Add | R^2^ (%) | LOD | Add | R^2^ (%) | LOD | Add | R^2^ (%) | LOD | Add | R^2^ (%) | LOD | Add | R^2^ (%) |
| PH14 |  |  |  |  |  |  |  |  |  |  |  |  |  |  |  |  |  |  |
| PH15 |  |  |  |  |  |  | 4.9 | -2.46 | 19.0 |  |  |  | 4.1 | -2.22 | 16.3 |  |  |  |
| PH16 | 3.6 | 1.35 | 14.5 | 4.64 | -1.75 | 18.3 |  |  |  | 5.8 | -1.66 | 22.4 | 5.8 | -1.66 | 22.4 | 5.0 | 1.64 | 19.7 |
| Allele conferring low plant height | L ^c^ | | | A | | | A | | | A | | | A | | | L | | |

^a^ Additive effect. ^b^ Percent of phenotypic variance explained by QTL. ^c^ A: Arve. L: Lavrans.
